# Transcriptomic markers of fungal growth, respiration and carbon-use efficiency

**DOI:** 10.1093/femsle/fnab100

**Published:** 2021-08-02

**Authors:** Fahri A Hasby, Florian Barbi, Stefano Manzoni, Björn D Lindahl

**Affiliations:** Department of Soil and Environment, Swedish University of Agricultural Sciences, Uppsala SE-75007, Sweden; Institute of Microbiology of the Czech Academy of Sciences, Vídeňská 1083, 14220 Praha 4, Czech Republic; Department of Physical Geography and Bolin Centre for Climate Research, Stockholm University, Svante Arrhenius väg 8, Stockholm, Sweden; Department of Soil and Environment, Swedish University of Agricultural Sciences, Uppsala SE-75007, Sweden

**Keywords:** growth, respiration, carbon-use efficiency, metatranscriptomics, gene markers, fungi

## Abstract

Fungal metabolic carbon acquisition and its subsequent partitioning between biomass production and respiration, i.e. the carbon-use efficiency (CUE), are central parameters in biogeochemical modeling. However, current available techniques for estimating these parameters are all associated with practical and theoretical shortcomings, making assessments unreliable. Gene expression analyses hold the prospect of phenotype prediction by indirect means, providing new opportunities to obtain information about metabolic priorities. We cultured four different fungal isolates (*Chalara longipes*, *Laccaria bicolor*, *Serpula lacrymans* and *Trichoderma harzianum*) in liquid media with contrasting nitrogen availability and measured growth rates and respiration to calculate CUE. By relating gene expression markers to measured carbon fluxes, we identified genes coding for 1,3-β-glucan synthase and 2-oxoglutarate dehydrogenase as suitable markers for growth and respiration, respectively, capturing both intraspecific variation as well as within-strain variation dependent on growth medium. A transcript index based on these markers correlated significantly with differences in CUE between the fungal isolates. Our study paves the way for the use of these markers to assess differences in growth, respiration and CUE in natural fungal communities, using metatranscriptomic or the RT-qPCR approach.

## Teaser

Development of genetic markers for fungal growth, respiration and carbon-use efficiency assessment.

## INTRODUCTION

Upon uptake, metabolic carbon is partitioned between biomass growth and respiration, with carbon-use efficiency (CUE) defined as the share of acquired carbon incorporated into growing biomass (Geyer *et al*. 2016; Manzoni *et al*. 2018). High CUE of microbial (in particular fungal) decomposers means lower proportional carbon losses during decomposition and higher accumulation of microbial biomass, which could enhance microbial-derived carbon storage in soils (Cotrufo *et al*. 2013; Wang *et al*. 2021). On the other hand, a high CUE can promote microbial proliferation, leading to more efficient plant litter exploitation with associated organic matter losses and lower plant-derived carbon storage (Allison, Wallenstein and Bradford 2010). Information about growth rates, respiration and CUE of microbial decomposers is, thus, essential in trait-based microbial modelling (Allison 2012; Manzoni *et al*. 2017; Zhang *et al*. 2018). However, direct empirical assessment of growth is challenging, since it depends on repeated measurements and intrusive disturbance. As a consequence, estimates of CUE are sensitive to the chosen methodology, and indirect short-term assessments may prove useful. For example, isotope-labeled substrates or calorespirometry have been used to assess growth and CUE in laboratory incubations (Geyer *et al*. 2019). CUE data on fungal decomposers is particularly limited, motivating our focus on fungi in this contribution.

‘Omics’ approaches, made possible by recent advances in sequencing technology, are becoming more common and are transforming research on fungal ecology (Nilsson *et al*. 2019). Variations in growth, respiration and CUE have a genetic basis, but are more likely related to differences in gene expression rather than gene presence and diversity (Barbi *et al*. 2020). Therefore, gene expression data may provide information about fungal metabolic traits, as mRNA sequences can be linked to specific metabolic conversions (Treseder and Lennon 2015). Genetic markers might even be used to assess differences in fungal traits in complex, natural fungal communities, using metatranscriptomic or RT-qPCR approaches (Kuske *et al*. 2015).

Genes coding for enzymes that facilitate polymerization of cell wall components such as glycosyl transferases (GT families in the CAZyme classification, Lombard *et al*. 2014) are particularly interesting targets for markers. Chitin synthase (GT2) and 1,3-β-glucan synthase (GT48) expression can be expected to be directly linked to fungal growth, since these enzymes are involved in the synthesis of chitin and β-glucan (Kelly *et al*. 1996; Sreenivasaprasad, Burton and Wood 2000), which are main components of fungal cell walls (Bowman and Free 2006). The tricarboxylic acid (TCA) cycle, which is central to aerobic metabolism and a hub of several catabolic and anabolic pathways, contains many different potential gene markers for respiration. Both NAD-dependent and NADP-dependent isocitrate dehydrogenase catalyse oxidative decarboxylation of isocitrate to 2-oxoglutarate and release CO_2_ (Haselbeck and McAlister-Henn 1993; Gálvez and Gadal 1995). Consecutively, the 2-oxoglutarate dehydrogenase complex catalyses oxidative decarboxylation of 2-oxoglutarate to succinyl-coenzyme A and CO_2_ (Repetto and Tzagoloff 1989). If useful transcriptional markers for growth and respiration may be identified, the expression ratio of these markers should reflect CUE. However, in contrast to genes coding for extracellular enzymes, for which the relationship between gene expression and observed process may seem relatively straightforward (Lindahl and Kuske 2013), relationships between gene expression and growth and respiration need to be verified under controlled conditions. In order to qualify as suitable, general markers, relationships between gene expression and phenotype have to be consistent across a variety of fungal species and in various environments.

Here, we devised a laboratory study where differences in growth rates, respiration and CUE among four fungal isolates and between contrasting growing conditions (high and low nitrogen availability) were related to gene expression data, with the aim to identify transcriptional markers that may be used to assess inter and intraspecific phenotypic variation. We specifically targeted a set of markers linked to growth and respiration, and evaluated these markers in relation to other potential marker genes. We hypothesized that:

1. Transcription of genes coding for glycosyl transferases that are active in polymerization of fungal cell wall components would correlate with relative mycelial growth rate.
2. Transcription of genes coding for enzymes in the TCA cycle would correlate with fungal respiration.
3. The transcription ratio of selected glycosyl transferase genes over TCA cycle genes would correlate with CUE.
4. That the above correlations would be valid both among fungal isolates as well as within isolates under different environmental conditions.

## METHODS

### Culture experiment

A total of four fungal isolates with sequenced genomes were selected to represent various fungal classes and life strategies: *Trichoderma harzianum* Rifai—an opportunistic mycoparasite (Weindling 1932) in the class Sordariomycetes, *Chalara longipes* (Preuss) Cooke—an ubiquitous colonizer of needle litter (Koukol 2011) in the class Leotiomycetes, *Lacaria bicolor* (Maire) P. D. Orton—an ectomycorrhizal fungus (Martin *et al*. 2008) in the order Agaricales (Agaricomycetes) and *Serpula lacrymans* (Wulfen)—a brown-rot wood decomposer (Kauserud *et al*. 2007) in the order Boletales (Agaricomycetes).

Fungal stock cultures were kept on Modified Melin-Norkrans (MMN) agar (Marx 1969) at room temperature in darkness. To produce agar free inoculum, colonized agar plugs were floated on the surface of liquid MMN medium, and extending mycelium was separated from the agar, further cultivated in liquid MMN for 1 week and macerated in the growth medium using an Ultra-Turrax (IKA, Germany). The concentration of mycelium in the inoculum was established by weighing the mycelial content of 2 mL of inoculum after drying at 40°C for 24 h (*N* = 10 for each isolate), and ranged 2–10 mg/mL.

To establish phenotypic variation in growth, respiration rate and CUE, the isolates were cultivated at two different conditions of nitrogen availability: liquid MMN medium with only glucose as the carbon source was modified to C:N ratios of 22 (N rich) or 221 (N poor) by altering the content of (NH_4_)_2_HPO_4_. The experimental system consisted of 50 mL of medium autoclaved in 250 mL Schott Duran® Bottles (DWK Life Sciences, Germany). Two connectors in the lid were attached to sterile syringe filters (0.2 µm; VWR, Radnor, Pennsylvania), to allow gas exchange. Systems were inoculated by adding 0.5 mL of mycelial macerate (1-5 mg of mycelium). Each treatment included nine replicates and one negative control (without inoculation). In total, 72 systems (four isolates × two growth media × nine replicates) were set up, and due to logistic constraints, the four isolates were assessed sequentially.

Glucose concentration in the medium was measured at regular intervals using a GM-100 glucose monitoring system (BioReactor Sciences, Lawrenceville, Georgia). The systems were harvested when roughly 30% of the glucose in the medium had been consumed (Figure S1 and Table S1, Supporting Information). Immediately before harvest, respiration was measured using an EGM-4 portable infra-red gas analyser (PP Systems, Amesbury, Massachusetts) with a closed sampling loop. Prior to measurement, the systems were flushed with filtered air for 1 h to allow dissolved CO_2_ to equilibrate with the atmosphere. CO_2_ accumulation was measured during 2.5 min for each system.

Mycelium was harvested by filtration through Whatman filter paper; ø 55m, pore size 12 mm (ThermoFisher Scientific, Waltham, Massachusetts) and immediately shock-frozen with liquid nitrogen, freeze dried, weighted using an ES120A analytical balance (Precisa Gravimetrics, Switzerland) and stored at –80°C. Total RNA was extracted from the harvested mycelium using the RNA mini kit (Qiagen, Germany) and cleaned from remaining DNA by the DNAse I kit (Sigma-Aldrich®, St. Louis, Missouri). Poly-A selection and mRNA library preparation was conducted using the TruSeq library preparation kit (Illumina, San Diego, California). Libraries were sequenced on the Illumina NovaSeq 6000 SP platform, yielding 50 bp paired-end sequences. Poly-A selection, library preparation and sequencing were performed by the SNP&SEQ Technology Platform of SciLifeLab, Uppsala, Sweden.

### Calculations

Relative growth rates (µ, day^–1^) at the time of harvest were estimated by the following equation under the assumption that growth was exponential,

(1) $$\begin{eqnarray*}
\mu = \frac{{\ln \left( {\frac{{{B_t}}}{{{B_0}}}} \right)}}{t},
\end{eqnarray*}$$

where *B_t_* is biomass at harvest, *B*_0_ is the amount of added inoculum, and *t* is number of days in culture.

Measured increases in CO_2_ concentration (ppm) over 2.5 min was converted to respiration rates (mmol carbon/day) using the ideal gas law with 1 atm air pressure, 20°C temperature and a 274 cm^3^ sampling loop volume. The C content of harvested mycelium was calculated based on assumed mycelial carbon content of 0.43 g carbon/g dry mass and then converted to mmol carbon (Zhang and Elser 2017). The metabolic quotient (qCO_2_, day^–1^) was calculated by dividing respiration rate measured immediately before harvest with the mass of the harvested mycelium and expressed in units of day^–1^. The CUE was calculated as the ratio between relative growth rate and the sum of relative growth rate and metabolic quotient,

(2) $$\begin{eqnarray*}
CUE = \frac{\mu }{{\mu + qC{O_2}}}.
\end{eqnarray*}$$

### Bioinformatic analyses

Raw paired-end reads were subjected to quality control using FastQC (Andrews *et al*. 2010). Sequencing adapter trimming and removal of low quality bases were performed in the program ‘Trimmomatic’ (Bolger, Lohse and Usadel 2014) with default settings. Reference genomes and gene annotations of *T. harzianum* (Druzhinina *et al*. 2018), *C. longipes* (Barbi *et al*. 2020), *L. bicolor* (Martin *et al*. 2008) and *S. lacrymans* (Eastwood *et al*. 2011) were retrieved from the JGI—Mycocosm database. Filtered mRNA sequences were mapped against respective genomes using ‘bowtie2’ (Langmead and Salzberg 2012) with default settings. Data was sorted, indexed, and converted to transcript count tables using ‘SAMtools’ (Li *et al*. 2009; Li 2011). Transcript data were normalized for gene lengths and sequencing effort according to the RPKM method (reads per kilo base per million mapped reads; Mortazavi *et al*. 2008). To enable analysis of expression of enzyme-encoding genes across fungal isolates, data was aggregated according to Enzyme Commission (EC) numbers, which denote a numerical classification of enzymes (Kanehisa 2017). The complete sequence was uploaded to NCBI-Sequence Read Archive (SRA; https://www.ncbi.nlm.nih.gov/sra) under the accession number PRJNA721630.

### Statistical analyses

All statistical analyses were performed in R version 3.6.2 (R Core Team 2019). Effects of isolates, medium and their interaction on relative growth rate, qCO_2_ and CUE, were evaluated by two-way ANOVA, with a post hoc Tukey test to evaluate differences between isolates. Due to large differences in variance between isolates, effects of growth medium were evaluated post hoc by *t*-tests for each isolate separately.

In order to evaluate gene markers for growth, we specifically targeted expression of 1,3-β-glucan synthase (EC 2.4.1.34) and chitin synthase (EC 2.4.1.16) encoding genes (Table S2, Supporting Information). We established a linear model with relative growth rate as response variable and expression of 1,3-β-glucan synthase or chitin synthase encoding genes (paralogs aggregated) as explaining variables, using the ‘lm’ function in the ‘stats’ package of R. Both response and explaining variables were log transformed. The same approach was applied to evaluate gene markers for respiration. We specifically targeted the expression of NAD-dependent isocitrate dehydrogenase (EC 1.1.1.41), NADP-dependent isocitrate dehydrogenase (EC 1.1.1.42) and 2-oxoglutarate dehydrogenase (EC 1.2.4.2) encoding genes (Table S2, Supporting Information), and established linear models with qCO_2_ as response variable and gene expression levels of either of the three enzymes classes as explaining variables.

A gene index (CUE_gene_) was calculated from coefficients and intercepts of linear models relating the selected gene markers to *μ* and qCO_2_. The linear models were defined as,

(3) $$\begin{eqnarray*}
\log \mu = {\alpha _1} + {\beta _1}\log GT48 + \varepsilon
\end{eqnarray*}$$

(4) $$\begin{eqnarray*}
\log qC{O_2} = {\alpha _2} + {\beta _2}\log KGD + \varepsilon,
\end{eqnarray*}$$

where α is the intercept, β is the slope coefficient for the explaining variable and ε represent the residuals; GT48 is the expression of 1,3-β-glucan synthase, and KGD is the expression of 2-oxoglutarate dehydrogenase. After rearranging and assuming ε = 0, Eqs. (3) and (4) can be expressed as,

(5) $$\begin{eqnarray*}
\mu = {e^{{\alpha _1}}}GT{48^{{\beta _1}}}
\end{eqnarray*}$$

(6) $$\begin{eqnarray*}
qC{O_2} = {e^{{\alpha _2}}}KG{D^{{\beta _2}}}.
\end{eqnarray*}$$

Thus, CUE_gene_ was defined as,

(7) $$\begin{eqnarray*}
CU{E_{gene}} = \frac{{{e^{{\alpha _1}}}GT{{48}^{{\beta _1}}}}}{{{e^{{\alpha _1}}}GT{{48}^{{\beta _1}}} + {e^{{\alpha _2}}}KG{D^{{\beta _2}}}}}.
\end{eqnarray*}$$

The gene index was evaluated as a predictor of measured CUE by linear regression. The *a priori* selected markers for growth and respiration were evaluated against other potential gene markers. All EC categories with an aggregated gene expression level of at least 10 RPKM in all samples were included, resulting in a list of 431 enzyme classes. Pearson's correlation coefficients between log-transformed expression levels of individual enzyme classes and log-transformed relative growth rate or qCO_2_ were calculated with the ‘cor’ function of the ‘stats’ R package.

To verify that fungi were nitrogen limited in the nitrogen poor medium, we investigated the expression of glutamine synthetase (EC 6.3.1.2), which is a central enzyme in ammonia assimilation (Montanini *et al*. 2003), as a marker. Two-way ANOVA was conducted to assess effects of isolates, medium and their interaction on expression of selected gene markers (log transformed) as well as on the CUE_gene_ index with post hoc Tukey tests to evaluate differences between isolates. Post hoc *t*-tests of effects of growth medium were conducted for each isolates separately, due to large differences in variance between isolates. The complete dataset used in the analyses was uploaded to Dryad reository (doi:10.5061/dryad.pvmcvdnkm).

## RESULTS AND DISCUSSION

### Sequencing and alignment

Transcriptome sequencing was successful for 65 of the 72 samples. One replicate of *C. longipes* was removed as an outlier due to very low expression of 1,3-β-glucan synthase and one replicate each of *L. bicolor* and *S. lacrymans* were removed due to negative relative growth rates. Thus, all statistical analyses were based on 62 observations. In total, 408 million Illumina sequences (96%) of *C. longipes*, 477 million sequences (95%) of *L. bicolor*, 487 million sequences (93%) of *S. lacrymans* and 378 million sequences (97%) of *T. harzianum* passed quality control. Of these, 316 million sequences (77%) mapped to the *C. longipes* genome, 374 million sequences (78%) mapped to the *L. bicolor* genome, 344 million sequences (71%) mapped to the *S. lacrymans* genome and 212 million sequences (56%) mapped to the *T. harzianum* genome.

### Growth markers

**Figure 1.**
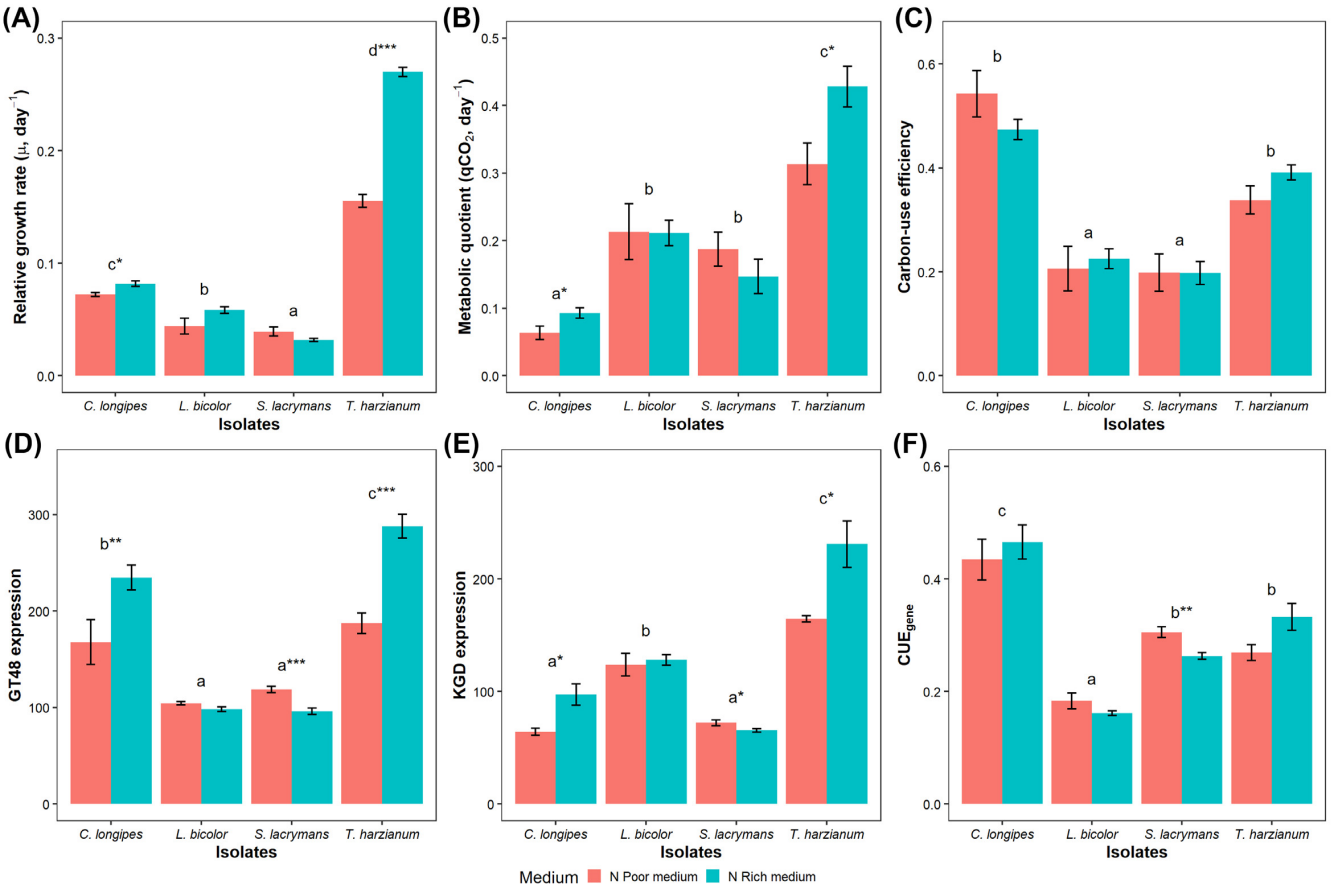
Effect of poor (red) vs. rich (blue) medium on: **(A)** relative growth rate, **(B)** metabolic quotient (qCO_2_), **(C)** carbon-use efficiency, **(D)** 1,3-β-glucan synthase (GT48) expression, **(E)** 2-oxoglutarate dehydrogenase (KGD) expression and **(F)** gene expression index of carbon-use efficiency (CUE_gene_) for different fungal isolates. Bars and whiskers indicate means ± SE. Different letters indicate statistically significant differences between isolates and asterisks (*P < 0.05; **P < 0.01 and ***P < 0.001) indicate statistically significant differences between medium (within isolates). Gene expression levels were RPKM normalized.

For *C. longipes* and *L. bicolor* the rates of glucose consumption were increasing, as indicated by downward concavity in the time trajectories, supporting our assumption of exponential growth. This was also the case for *T. harzianum* in rich medium, whereas in poor medium growth was retarded towards the end of the culture period, presumably due to N limitation. Glucose consumption by *S. lacrymans* was more irregular and with a linear trend (Figure S1, Supporting Information). Biomass at harvest ranged from 1.3 to 54 mg, with *T. harzianum* having the highest relative growth rate, followed by *C. longipes*, *L. bicolor* and *S. lacrymans* (Fig. 1A). There was a significant interaction effect of isolate and medium on growth rate (Table 1) with *T. harzianum* and *C. longipes* growing significantly faster in the rich medium.

**Figure 2.**
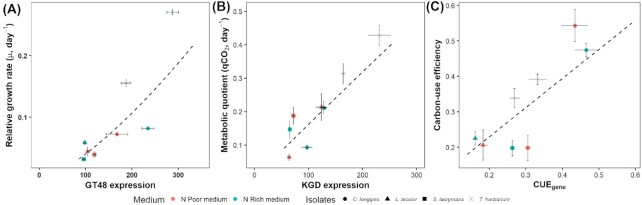
Relationships between **(A)** growth rate and 1,3-β-glucan synthase (GT48) expression, **(B)** qCO_2_ and 2-oxoglutarate dehydrogenase (KGD) expression and **(C)** measured carbon-use efficiency and gene index (CUE_gene_) of four different fungal isolates grown in two different media. Symbols represent means ± SE and gene expression levels were RPKM normalized. Dashed line represents linear model presented in Table 1.

**Table 1.** Two-way ANOVA table of C dynamics.

	Log growth rate (mmol C/day)					Log metabolic quotient (qCO_2_, day^–1^)					Log carbon-use efficiency				
	Sum Sq	Df	*F* value	Pr(>F)	R^2^	SumSq	Df	*F*value	Pr(>F)	R^2^	Sum Sq	Df	*F*value	Pr(>F)	R^2^
Isolates	27.38	3	143.43	0***	0.90	17.39	3	45.64	0***	0.73	0.90	3	46.44	0***	0.73
Medium	0.66	1	10.44	0.0021**		0.21	1	1.61	0.21		9 × 10^–5^	1	0.014	0.91	
Isolates : Medium	1.17	3	6.12	0.0012**		0.96	3	2.52	0.067		0.026	3	1.36	0.26	
Residuals	3.44	54				6.86	54				0.35	54			
	Log GT48					Log KGD					CUE_gene_				
	Sum Sq	Df	Fvalue	Pr(>F)	R^2^	Sum Sq	Df	Fvalue	Pr(>F)	R^2^	Sum Sq	Df	Fvalue	Pr(>F)	R^2^
Isolates	8.55	3	171.10	0***	0.92	9.83	3	89.92	0***	0.85	7.37	3	104.29	0***	0.86
Medium	0.14	1	8.38	0.0055**		0.30	1	8.22	0.0059**		0.0027	1	0.11	0.74	
Isolates : Medium	1.11	3	22.21	0***		0.55	3	5.06	0.0037**		0.29	3	4.13	0.011*	
Residuals	0.90	54				1.97	54				1.27	54			
															
	Log GS														
	Sum Sq	Df	Fvalue	Pr(>F)	R^2^										
Isolates	8.32	3	36.19	0***	0.73										
Medium	4.47	1	58.36	0***											
Isolates : Medium	0.64	3	2.77	0.051^+^											
Residuals	4.14	54													

Notes: + *P* < 0.10; *P < 0.05; **P < 0.01 and ***P < 0.001.

Gene expression of both 1,3-β-glucan synthase and chitin synthase explained a significant fraction of the total variation in relative growth rate. However, 1,3-β-glucan synthase expression was a much better predictor of relative growth across isolates and medium (R^2^ = 0.64) than chitin synthase expression (R^2^ = 0.09), and was chosen as the best marker (Table 2 and Fig. 2A ). For comparison, an ANOVA in which relative growth rate was predicted by isolate, medium and their interaction had an R^2^ of 0.90 (Table 1). 1,3-β-glucan synthase expression was significantly different between isolates (*P* < 0.001) with higher expression in the faster growing *T. harzianum* and *C. longipes* than in the slow-growing *L. bicolor* and *S. lacrymans* (Fig. 1D). There was a significant interaction between the effects of isolates and medium on 1,3-β-glucan synthase expression (Table 1) with higher 1,3-β-glucan synthase expression in rich medium for *T. harzianum* and *C. longipes* but higher expression in poor medium for *S. lacrymans*. When comparing correlation with growth across all enzyme-encoding genes, 1,3-β-glucan synthase ranked 14th out of 431 enzyme classes (Fig. 4A). The 13 enzyme classes that exhibited stronger correlations with growth rate than1,3-β-glucan synthase are listed in Table S3 (Supporting Information), and were mainly related to glycolysis, cell membrane synthesis and post-translational modification of proteins.

**Table 2.** Linear model output of different genes and gene index.

Dependent variable	Explaining variable	Intercept	β	R^2^	Residual SE (df = 60)	*F* statistic (df = 1; 60)
Log relative growth rate (µ, day^–1^)	Log GT48	–9.64***	1.40***	0.64	0.45	107.46***
	Log GT2	–6.37***	0.63*	0.09	0.71	6.02*
Log metabolic quotient (qCO_2_, day^–1^)	Log KGD	–6.32***	0.98***	0.48	0.47	55.69***
	Log IDH_NAD_	0.20	–0.15	0.02	0.64	1.12
	Log IDH_NADP_	0.22***	0.12	0.01	0.65	0.50
Carbon-use efficiency	CUE_gene_	0.07	0.82***	0.42	0.11	43.24***

Notes: + *P* < 0.10; *P < 0.05; **P < 0.01 and ***P < 0.001.

**Figure 3.**
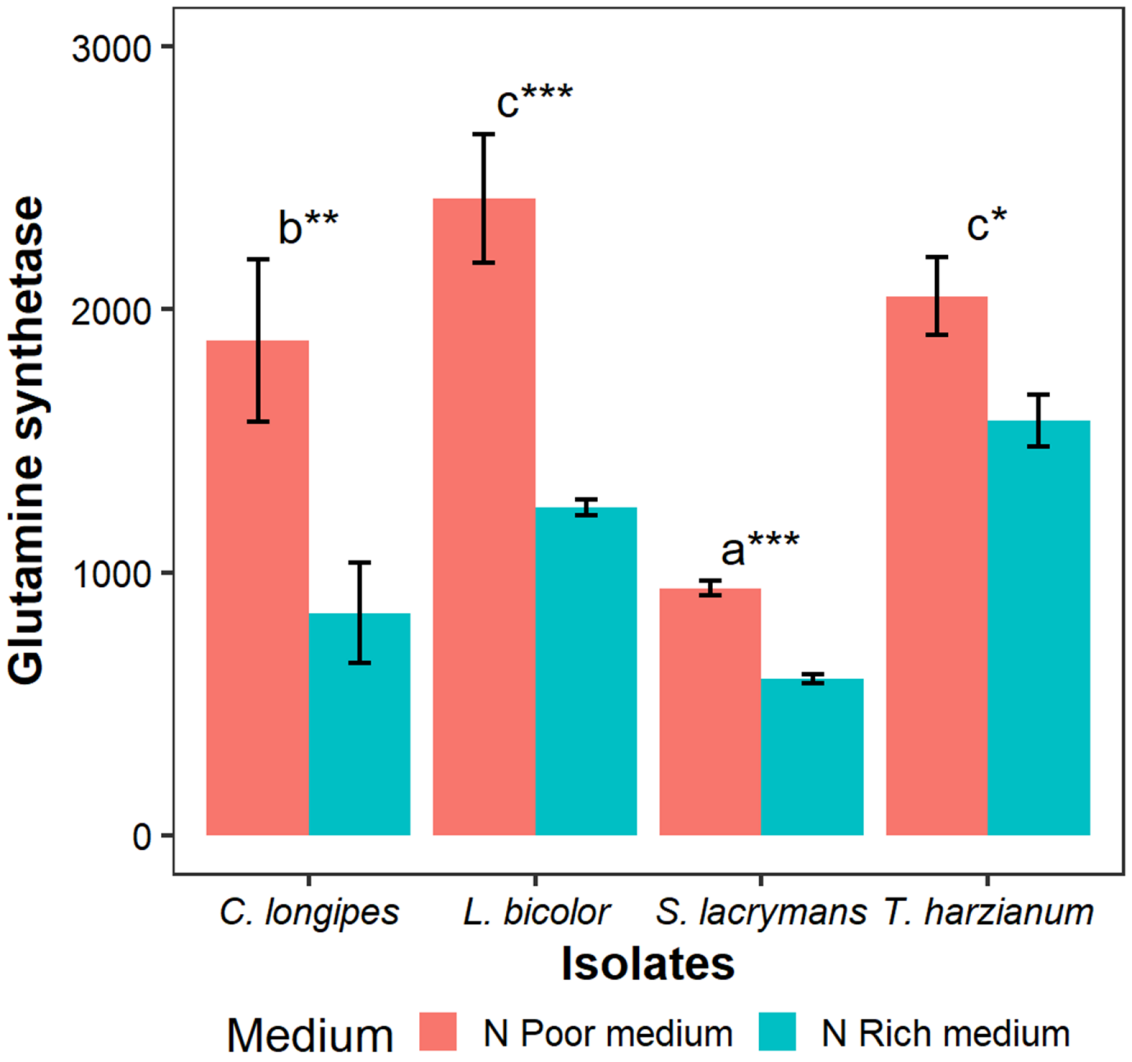
Effect of poor (red) vs. rich (blue) medium on glutamine synthetase expression of different fungal isolates. Bars and whiskers indicate means ± SE. Different letters indicate statistically significant differences between isolates and asterisks (*P < 0.05; **P < 0.01 and ***P < 0.001) indicate statistically significant differences between media (within isolates). Gene expression levels were RPKM normalized.

The relative growth rates of *C. longipes* and *T. harzianum* (ascomycetes) were higher than those of *L. bicolor* and *S. lacrymans* (basidiomycetes), and in spite of the very high C:N ratio of the poor medium (Fig. 1A), growth was supressed by nitrogen limitation only for the two most rapidly growing isolates. Nitrogen limitation was, however, apparent for all isolates in the consistent upregulation of glutamine synthetase (EC: 6.3.1.2) in poor medium (Fig. 3), which is required to maintain ammonium transport across the cell membrane when external ammonium concentrations are low (Kershaw and Stewart 1989). Our results show that 1,3-β-glucan synthase expression reflects the variation of growth rate between media (across environments) and among isolates (across taxa). At the mycelial level, the relative proportion of 1,3-β-glucan synthase mRNA in the transcriptome may reflect the proportion of growing tip cells as well as the rate of growth of these cells. At this scale, morphological differences, such as hyphal branching frequency might be a more important determinant of relative growth rates than metabolic differences at the cellular level.

The significant negative intercept in the linear model of growth predicted by 1,3-β-glucan synthase expression (Table 2) implies a basal expression of 1,3-β-glucan synthase (Fig. 2A; *x*-axis intercept at x = 6.89), possibly linked to cell walls maintenance in non-growing cells, balanced by continuous turnover of cell wall material. In addition, the rate of cell wall assembly (growth) could be hampered by restricted allocation of sugars for growth (i.e. substrate limitation rather than enzyme limitation). However, it is also possible that growth responds to gene transcription in a logarithmic manner. Although our results represent a wide range of growth rates, linearity along the full range of variation present under natural conditions remains to be confirmed.

In this study, the expression of 1,3-β-glucan synthase reflected short-term relative investment in growth at harvest (i.e. the ‘intention’ of the fungi to grow). For this reason, we expected that expression of this gene would correlate with the relative growth rate at the time of harvest, as approximated by Eq. (3). In future studies, isotope labeled substrate added shortly before harvest might help to elucidate the relation between carbon uptake and short-term realized growth. We also acknowledge that there growth rate might be underestimated due to uncertainties in the amount of added inoculum, since the added macerate might have contained both active cells and dead tissues. A lag phase before growth might have commenced after inoculation, contributing additional uncertainty, particularly for *S. lacrymans*, which took a long time to start growing after inoculation and exhibited low estimated growth relative to 1,3-β-glucan synthase expression. However, exponential growth models do account for a possible initial slow growth rate (they assume time invariant *specific* growth rate), thus reducing the negative consequences of a lag phase.

Apart from cell proliferation and elongation, 1,3-β-glucan synthase has been proposed as a marker for stress response, since the 1,3-β-glucan production is involved in cell fortification to protect from environmental stressors (Treseder and Lennon 2015). However, this might also be considered as part of growth, since thicker cell walls have higher mass.

### Respiration markers

The qCO_2_ was significantly different between isolates (*P* < 0.001), with highest values in *T. harzianum*, intermediate values in *L. bicolor* and *S. lacrymans* and lowest values in *C. longipes* (Fig. 1B), and a marginally significant isolates x medium interaction effect (*P* = 0.07), with *C. longipes* and *T. harzianum* having significantly higher qCO_2_ in rich medium (Table 1). This is contrary to previous observations of a negative relationship between qCO_2_ and nitrogen availability in complex microbial communities in soils (Riggs *et al*. 2015; Spohn 2015; Spohn *et al*. 2016). The discrepancy may be ascribed to the artificial conditions of the pure culture system, for which it has been speculated that fast growing isolates may suffer higher metabolic costs of growth and cell maintenance (Lipson 2015).

**Figure 4.**
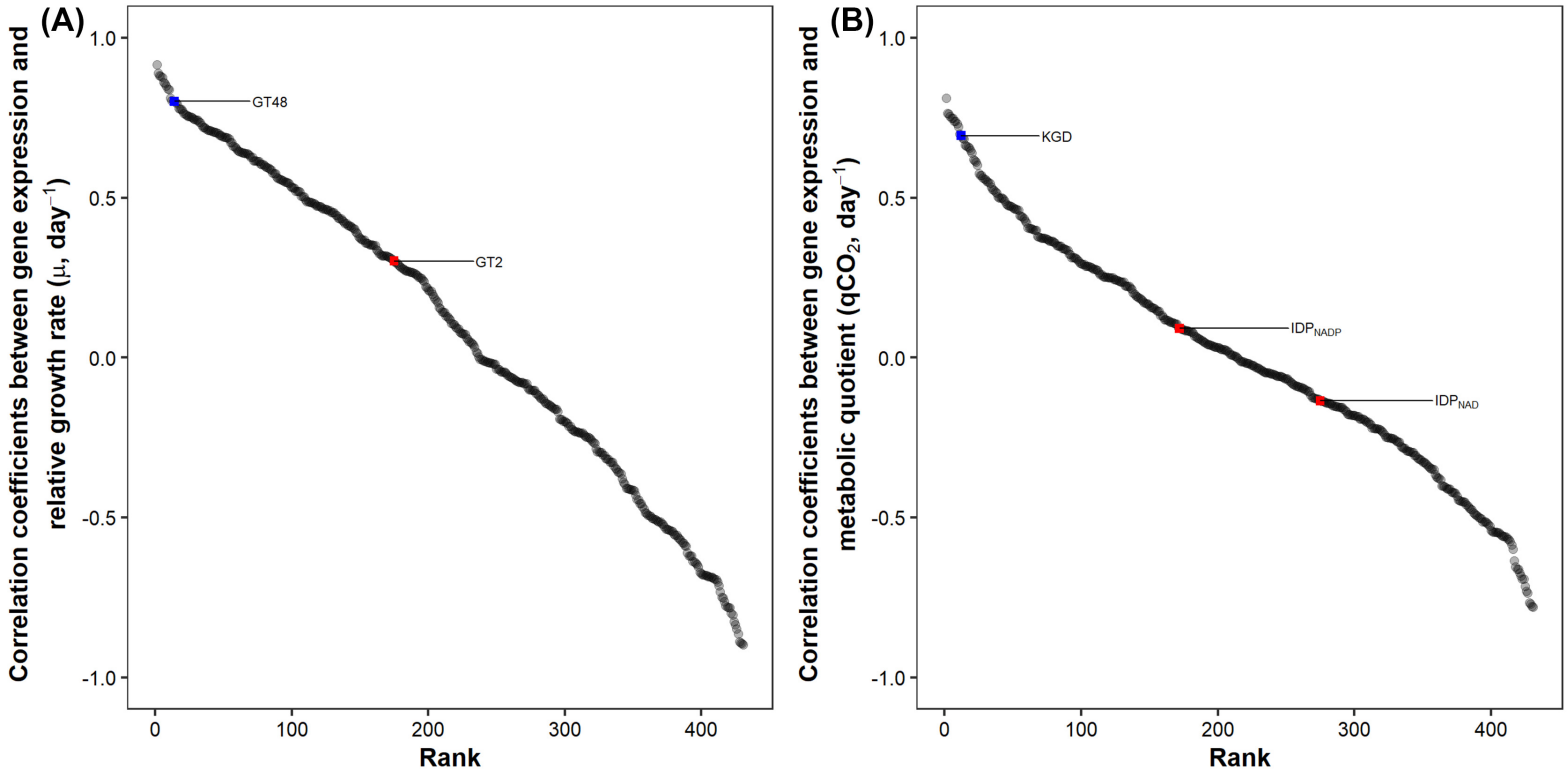
Ranked Pearson's correlation coefficients of all log-transformed enzyme encoding genes with log-transformed growth rate **(A)** and qCO_2_**(B)**. The blue square represents the marker selected to be used in CUE_gene_ and the red square represents marker(s) not selected.

Among gene candidates in the TCA cycle, only the 2-oxoglutarate dehydrogenase gene expression explained significant (*P* < 0.001) variation in qCO_2_ across isolated and medium composition, achieving R^2^ = 0.48 (Fig. 2B and Table 2). For comparison, a two-way ANOVA in which qCO_2_ was predicted by isolate, medium and their interaction had R^2^ = 0.73 (Table 1). 2-oxoglutarate dehydrogenase expression was significantly different between isolates with higher expression in *T. harzianum* and *L. bicolor* and lower in *S. lacrymans* and *C. longipes* (Fig. 1E). There was a significant isolates x medium interaction effect with higher 2-oxoglutarate dehydrogenase expression for *T. harzianum* and *C. longipes* in rich medium but slightly lower expression in poor medium for *S. lacrymans* (Table 2). Similar to 1,3-β-glucan synthase, we observed a significant negative intercept in the linear model of expression (Table 2), indicating baseline expression (Fig. 2B; *x*-axis intercept at x = 6.32). Constitutive expression of this genes may not result in CO_2_ production, if the TCA cycle is constrained by other regulatory bottlenecks. When comparing correlation with qCO_2_ across all enzyme encoding genes, 2-oxoglutarate dehydrogenase ranked 12th out of 431 enzyme classes (Fig. 4B). The 11 enzyme classes that exhibited stronger correlations with growth rate than 2-oxoglutarate dehydrogenase are listed in Table S4 (Supporting Information) and were mainly related to glycolysis, pentose-phosphate pathway and post-translational modification of proteins.

### Gene index of CUE

Measured CUE was significantly different between isolates (*P* < 0.001; Table 1), with highest efficiency for *C. longipes*, followed by *T. harzianum* and lowest efficiency in *L. bicolor* and *S. lacrymans*, without any significant effect of culturing medium (Fig. 1C and Table 1). Based on the predictive models of relative growth and qCO_2_, a gene expression index for CUE was calculated as:

(8) $$\begin{eqnarray*}
CU{E_{gene}} = \frac{{{e^{ - 9.64}}GT{{48}^{1.4}}}}{{{e^{ - 9.64}}GT{{48}^{1.4}} + {e^{ - 6.37}}KG{D^{0.98}}}}.
\end{eqnarray*}$$

After rearranging, Eq. (8) can be expressed in a more compact form as,

(9) $$\begin{eqnarray*}
CU{E_{gene}} = {\left( {1 + 26.3\frac{{KG{D^{0.98}}}}{{GT{{48}^{1.4}}}}} \right)^{ - 1}}.\end{eqnarray*}$$

CUE_gene_ was significantly correlated with CUE, with R^2^ = 0.42 (Fig. 2C and Table 2). For comparison, an ANOVA in which CUE was predicted by isolate, medium and their interaction had R^2^ = 0.80 (Table 1). The higher fraction of explained variance in the latter statistical model is explained by its higher number of parameters (six parameters) compared to our simpler linear regression between CUE_gene_ and CUE (two parameters). CUE_gene_ was significantly different between isolates, with highest values in *C. longipes*, intermediate values in *T. harzianum* and *S. lacrymans* and lowest values in *L. bicolor*. There was a significant isolates x medium interaction effects on CUE_gene_ with a negative effect on *S. lacrymans* in rich medium (Fig. 1F).

We observed no significant effect of N availability on measured CUE. This finding is surprising given the high C:N ratio (220:1) of the nutrient poor medium—typically CUE decreases at high C:N ratio as microorganisms become nitrogen limited and excess carbon is eliminated or invested in non-growth processes (Manzoni *et al*. 2017). The lack of CUE response could be due to allocation adjustments in the face of nitrogen shortage that did not involve waste respiration (Camenzind *et al*. 2021). As carbon and nitrogen were supplied in uncoupled form in liquid media, the isolates could adjust the relative acquisition of different resources, as indicated by upregulation of glutamine synthetase gene expression. In contrast, CUE varied across isolates, and in particular, the two ascomycetes had significantly higher CUE than the two basidiomycetes. A similar pattern was also observed in our gene expression based assessment (CUE_gene_). The CUE_gene_ of *S. lacrymans* was intermediate and similar to *T. harzianum* with higher expression of 1,3-β-glucan synthase than expected from direct measurements of growth.

Although this index is probably not directly transferable to other studies (due to different bases for data normalization), the simple expression ratio of 1,3-β-glucan synthase over 2-oxoglutarate dehydrogenase was also a useful indicator of CUE (Figure S2, Supporting Information).

## CONCLUSIONS

Although we identify many potential genetic markers for growth and respiration, we see pedagogic values for the use of our *a priori* selected markers. 1,3-β-glucan synthase is directly active in polymerization of the main constituent of the fungal cell wall and its interpretation as a growth marker is intuitive. Similarly, 2-oxoglutarate dehydrogenase is directly involved in the generation of CO_2_ and, thus, an attractive marker for respiration. This study presents a basis for theoretical linkages of transcripts, growth rates, respiration and CUE under idealized conditions, but we see a potential utility of these markers in future assessment of differences in growth, respiration and CUE in natural fungal communities, using metatranscriptomics or RT-qPCR approaches.

## Supplementary Material

fnab100_Supplemental_files
