# Supplementary material for: Transcriptomic markers of fungal growth, respiration and carbon-use efficiency
Source: FEMS Microbiol Lett. 2021 Aug 2;368(15):fnab100. doi: 10.1093/femsle/fnab100 (PMC8374604; doi:10.1093/femsle/fnab100)
Supplement: fnab100_Supplemental_files [file fnab100_supplemental_files.docx]

**Supplementary information**

Table S1. Total culture period and average glucose consumption of all isolates in different media

| Isolates | Medium | Culture period | Glucose consumption  (Means ± SE) |
| --- | --- | --- | --- |
| C. *longipes* | N poor media | 26 days | 43.0 ± 1.8 % |
|  | N rich media |  | 58.2 ± 3.2 % |
|  |  |  |  |
| L. *bicolor* | N poor media | 23 days | 34.3 ± 2.8 % |
|  | N rich media |  | 35.2 ± 2.8 % |
|  |  |  |  |
| S. *lacrymans* | N poor media | 25 days | 16.9 ± 1.2 % |
|  | N rich media |  | 24.4 ±1.4 % |
|  |  |  |  |
| T. *harzianum* | N poor media | 7 days | 32.2 ± 1.5 % |
|  | N rich media |  | 80.5 ± 2.2 % |

Table S2. Gene marker candidates of different isolates

| Isolates | | Markers |  | Enzyme family | E.C. number | JGI transcript ID | JGI protein ID |
| --- | --- | --- | --- | --- | --- | --- | --- |
| C. *longipes* | | Growth |  | GT48 | 2.4.1.34 | 346190, 455219 | 346138, 455167 |
|  |  |  |  | GT2 | 2.4.1.16 | 327914, 328554, 376611, 377305, 378237, 426211, 539183, 546778 | 327862, 328502, 376559, 377253, 378185, 426159  539131, 546726 |
|  |  | Respiration |  | KGD | 1.2.4.2 | 352321 | 352269 |
|  |  |  |  | IDP_NAD_ | 1.1.1.41 | 539034, 550863 | 538982, 550811 |
|  |  |  |  | IDP_NADP_ | 1.1.1.42 | 435426 | 435374 |
|  |  | | | | | | |
| L. *bicolor* | | Growth |  | GT48 | 2.4.1.34 | 187667, 317105 | 187667, 317105 |
|  |  |  |  | GT2 | 2.4.1.16 | 181144, 186476, 187522, 188623, 239741, 246643, 247128, 380073, 676353, 689201 | 181144, 186476, 187522, 188623, 239741, 246643, 247128, 380073, 676353, 689201 |
|  |  | Respiration |  | KGD | 1.2.4.2 | 245492, 691668 | 245492, 691668 |
|  |  |  |  | IDP_NAD_ | 1.1.1.41 | 311842, 311861 | 311842, 311861 |
|  |  |  |  | IDP_NADP_ | 1.1.1.42 | 317084 | 317084 |
|  | |  |  |  |  |  |  |
| S. *lacrymans* | | Growth |  | GT48 | 2.4.1.34 | 87516, 172886 | 87516, 172886 |
|  |  |  |  | GT2 | 2.4.1.16 | 55348, 64710, 106295, 107038, 113672, 115801, 151765, 168131, 172317 | 55348, 64710, 106295, 107038, 113672, 115801, 151765, 168131, 172317 |
|  |  | Respiration |  | KGD | 1.2.4.2 | 123478 | 123478 |
|  |  |  |  | IDP_NAD_ | 1.1.1.41 | 177461, 177525 | 177461, 177525 |
|  |  |  |  | IDP_NADP_ | 1.1.1.42 | 51285 | 51285 |
|  |  | | | | | | |
| T. *harzianum* | | Growth |  | GT48 | 2.4.1.34 | 3225, 83485 | 3225, 83485 |
|  |  |  |  | GT2 | 2.4.1.16 | 1177, 1178, 2190, 84529, 85658, 95405, 131849 | 1177, 1178, 2190, 84529, 85658, 95405, 131849 |
|  |  | Respiration |  | KGD | 1.2.4.2 | 510673 | 510673 |
|  |  |  |  | IDP_NAD_ | 1.1.1.41 | 71098, 508954 | 71098, 508954 |
|  |  |  |  | IDP_NADP_ | 1.1.1.42 | 86285 | 86285 |

**
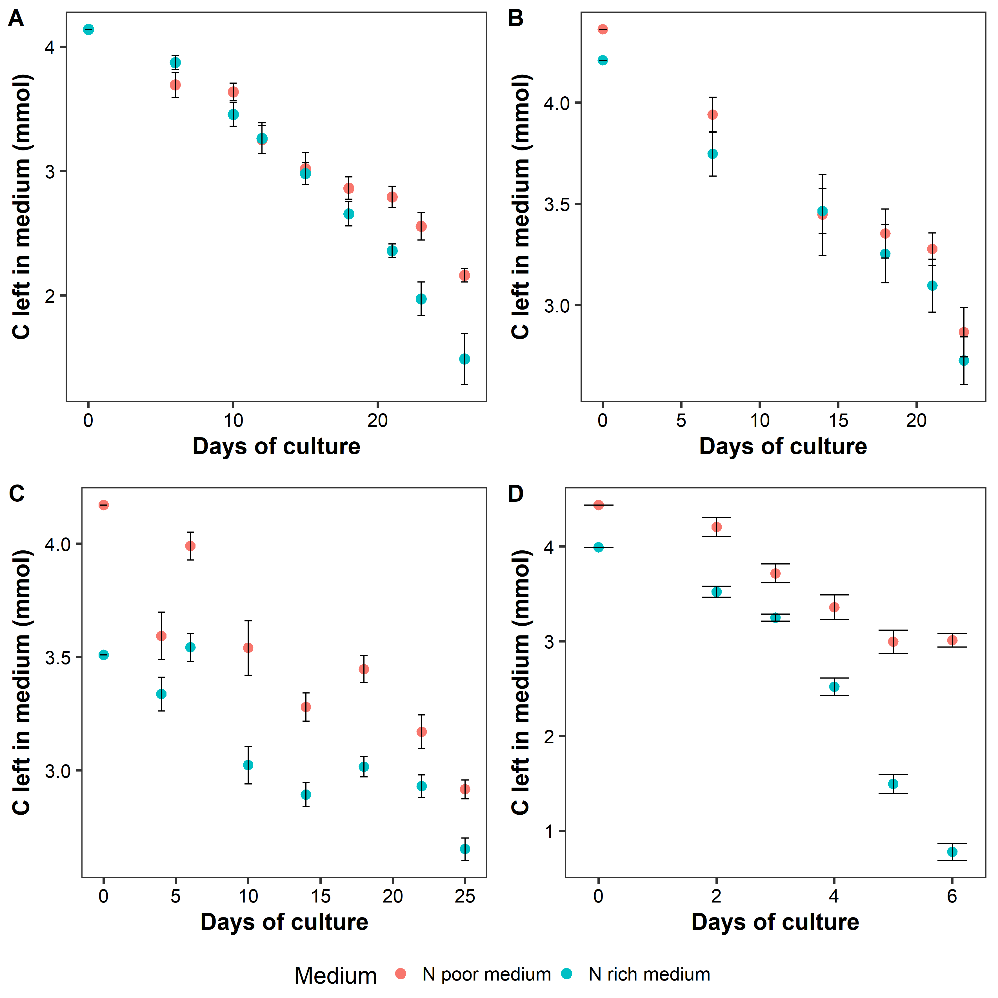
**

Figure S1. Glucose consumption of (A) *C. longipes*, (B) *L. bicolor*, (C) *S. lacrymans* and (D) *T. harzianum*  in two different media. Symbols represent means ± SE.


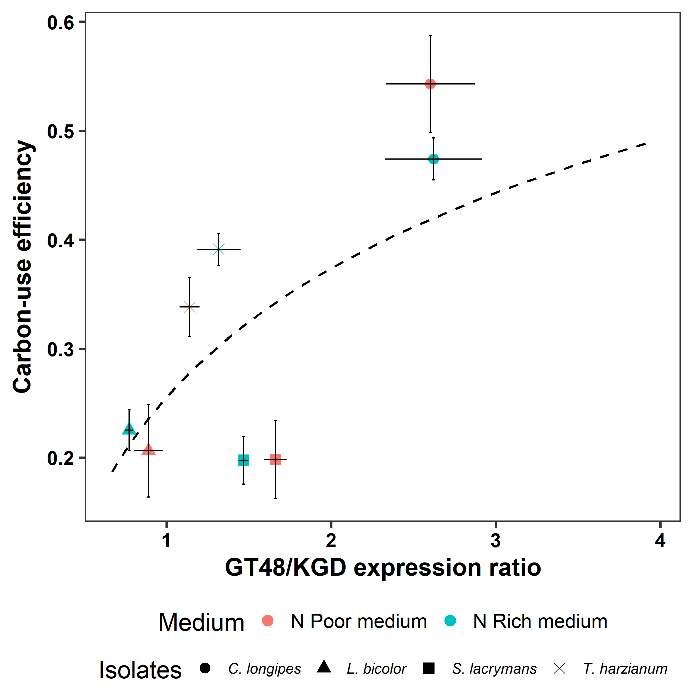


Figure S2. Relationships between measured carbon-use efficiency and expression ratio of 1,3-β-glucan synthase (GT48) over 2-oxoglutarate dehydrogenase (KGD) of four different fungal isolates grown in two different media. Symbols represent means ± SE and gene expression levels were RPKM normalized. Dashed line represents regression line on log-transformed expression ratio.

Table S3. Genes that ranked higher than 1,3-beta-glucan synthase in correlation to relative growth rate

| Rank | Enzyme | Pearson's correlation coefficient (r) | E.C. Number |
| --- | --- | --- | --- |
| 1 | Peptide alpha-N-acetyltransferase | 0.92 | 2.3.1.88 |
| 2 | Methionyl aminopeptidase | 0.89 | 3.4.11.18 |
| 3 | Phosphorylase | 0.88 | 2.4.1.1 |
| 4 | Ubiquitin thiolesterase | 0.88 | 3.1.2.15 |
| 5 | Glutamate--tRNA ligase | 0.88 | 6.1.1.17 |
| 6 | NAD(+) kinase | 0.86 | 2.7.1.23 |
| 7 | Cytochrome-c oxidase | 0.86 | 1.9.3.1 |
| 8 | Choline kinase | 0.85 | 2.7.1.32 |
| 9 | Di-trans-poly-cis-decaprenylcistransferase | 0.84 | 2.5.1.31 |
| 10 | 3-oxo-5-alpha-steroid 4-dehydrogenase | 0.84 | 1.3.99.5 |
| 11 | Phosphoglycerate kinase | 0.81 | 2.7.2.3 |
| 12 | Phosphotransferases (nitrogenous group acceptor) | 0.80 | 2.7.3.- |
| 13 | Serine--tRNA ligase | 0.80 | 6.1.1.11 |
| 14 | 1,3-beta-glucan synthase (GT48) | 0.80 | 2.4.1.34 |

Table S4. Genes that ranked higher than 2-oxoglutarate dehydrogenase in correlation to qCO_2_

| Rank | Enzyme | Pearson's correlation coefficient (r) | E.C. Number |
| --- | --- | --- | --- |
| 1 | Leucine--tRNA ligase | 0.81 | 6.1.1.4 |
| 2 | Cyclopropane-fatty-acyl-phospholipid synthase | 0.76 | 2.1.1.79 |
| 3 | Fructose-bisphosphate aldolase | 0.76 | 4.1.2.13 |
| 4 | Chitin synthase | 0.75 | 2.4.1.16 |
| 5 | Saccharopine dehydrogenase | 0.75 | 1.5.1.7 |
| 6 | Transaldolase | 0.75 | 2.2.1.2 |
| 7 | Licheninase | 0.74 | 3.2.1.73 |
| 8 | Guanosine-diphosphatase | 0.74 | 3.6.1.42 |
| 9 | Argininosuccinate synthase | 0.73 | 6.3.4.5 |
| 10 | CDP-diacylglycerol--serine O-phosphatidyltransferase | 0.72 | 2.7.8.8 |
| 11 | Exoribonuclease II | 0.70 | 3.1.13.1 |
| 12 | 2-oxoglutarate dehydrogenase (KGD) | 0.69 | 1.2.4.2 |
